# Glymphatic system in the thalamus, secondary degeneration area was severely impaired at 2nd week after transient occlusion of the middle cerebral artery in rats

**DOI:** 10.3389/fnins.2022.997743

**Published:** 2022-10-06

**Authors:** Chanchan Li, Luyi Lin, Chengfeng Sun, Xiaozhu Hao, Lekang Yin, Xiaoxue Zhang, Jiaqi Tian, Zhengwei Yao, Xiaoyuan Feng, Yanmei Yang

**Affiliations:** ^1^Department of Radiology, Huashan Hospital, Fudan University, Shanghai, China; ^2^Department of Radiology, Fudan University Shanghai Cancer Center, Shanghai, China; ^3^Department of Radiology, Zhongshan Hospital, Fudan University, Shanghai, China; ^4^Department of Radiotherapy, Shanghai Eastern Hepatobiliary Surgery Hospital, Shanghai, China; ^5^Department of Radiology, Renji Hospital, Shanghai Jiao Tong University, Shanghai, China

**Keywords:** ischemic stroke, glymphatic system, AQP4 polarization, astrogliosis, MRI, middle cerebral artery occlusion

## Abstract

**Background and objectives:**

The glymphatic system is a recently discovered cerebrospinal fluid transport system and little is known about its dynamic changes after stroke. This study aimed to dynamically observe the structural and functional changes of the impaired glymphatic system in the thalamus after ischemic stroke by pathology and MRI.

**Materials and methods:**

Ischemic stroke was induced by the middle cerebral artery occlusion (MCAO) model. A total of 20 Sprague-Dawley rats were randomly assigned into four groups: sham, MCAO 1 week, MCAO 2 week, and MCAO 2 month. All rats successively underwent neurological examination, dynamic contrast-enhanced MRI (DCE-MRI), and immunofluorescence staining. Immunofluorescence staining of glial fibrillary acidic protein (GFAP), aquaporin-4 (AQP4), ionized calcium-binding adaptor molecule 1 (Iba1), and beta-amyloid precursor protein (APP) were done in thalamus ventroposterior nucleus.

**Results:**

The astrocyte and microglial activation and the APP deposition in the MCAO 2 week group were the highest (*P* < 0.05 for all). The AQP4 polarization rates of the MCAO 2 week and 2 month groups were the lowest (*P* < 0.05 for all). Although there was no correlation between histological changes and MRI metrics in all four groups (*P* > 0.05 for all), the tendency of the APP deposition was nearly consistent with the one of the contrast agent retention in DCE-MRI.

**Conclusion:**

The glymphatic system in the thalamus was severely impaired at 2nd week after MCAO, and may be revealed by DCE-MRI. This study may provide a relevant theoretical basis for making a thorough inquiry of the mechanism of brain injury after stroke and clinical treatment of ischemic stroke and help readers appreciate the importance of DCE-MRI.

## Introduction

Stroke is the destruction of brain function and structure and is a disease associated with high mortality and disability worldwide (Shi et al., 2021; Wang et al., 2021; Zhou et al., 2021). Beta (β)-amyloid precursor protein (APP) is a transmembrane protein with a long extracellular N-terminal and short intracellular C-terminal domain (Kapasi et al., 2021). APP is widely expressed in the brain, where its abnormal upregulation can lead to the accumulation of β-amyloid (Aβ) (Hilal et al., 2018; Yu et al., 2019). Cerebral ischemia leads to upregulation and accumulation of Aβ (Kapasi et al., 2021). Soluble Aβ can be cleared from the brain through various mechanisms including enzymatic degradation, glial cell phagocytosis, transport across the blood-brain barrier, and glymphatic system clearance (Feng et al., 2020). The glymphatic system is a recently discovered cerebrospinal fluid (CSF) transport system. Through the perivascular space and aquaporin 4 (AQP4) on astrocytes, it promotes the exchange of CSF and interstitial fluid (ISF), clears brain metabolic waste, and maintains the stability of the internal environment within the brain (Wang et al., 2012; Rasmussen et al., 2018; Nedergaard and Goldman, 2020; Zhou et al., 2021). However, little is known about the dynamic changes in the structure and function of the impaired glymphatic system after ischemic stroke. Dynamic contrast-enhanced MRI (DCE-MRI), which captures the tissue’s signal enhancement after injection of a gadolinium-based contrast agent, is an effective method to evaluate the function of the glymphatic system *in vivo* (Lin et al., 2020; Breit et al., 2021; Lee et al., 2021; Ray et al., 2021).

We hypothesized that the dynamic changes in structure and function of the impaired glymphatic system after ischemic stroke could be observed by pathology and MRI. To test the hypothesis, in this study, immunofluorescence staining and intracisternal DCE-MRI were performed in the 1st week, 2nd week, and 2nd month after middle cerebral artery occlusion (MCAO), separately, to dynamically observe the structural and functional changes of the impaired glymphatic system in the thalamus, secondary degeneration area after ischemic stroke in rats. The results of this study may provide the theoretical basis for exploring new targets for ischemic stroke treatment and help readers appreciate the importance of DCE-MRI.

## Materials and methods

### Animals and stroke model

**FIGURE 1:**
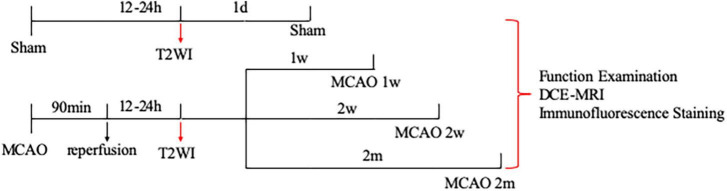
Study design of the experiment. A total of 20 rats were randomly assigned into four groups: sham group (*n* = 5), group of MCAO 1 week (*n* = 5), group of MCAO 2 week (2w) (*n* = 5), and group of MCAO 2 month (2m) (*n* = 5). The ischemic severity was checked by T2-weighted MRI 12–24 h after the operation. Thereafter, all rats successively underwent neurological examination, DCE-MRI, and immunofluorescence staining. To be specific, neurological examination, DCE-MRI with intrathecal Gd-DTPA administration, and immunofluorescence staining were performed on the 1st day after the operation for the sham group, in the 1st week after the operation for the group of MCAO 1 week rats, in the 2nd week after the operation for the group of MCAO 2 week rats, as well as in the 2nd month after the operation for the group of MCAO 2 month rats. MCAO, middle cerebral artery occlusion; DCE-MRI, dynamic contrast-enhanced MRI; Gd-DTPA, gadolinium-diethylenetriaminepentaacetic acid.

Adult male Sprague-Dawley rats (260–270 g) were obtained from Shanghai Jie Si Jie Laboratory Animal Co., Ltd. (Shanghai, China). A total of 20 rats were randomly assigned into four groups: sham group (*n* = 5), group of MCAO 1 week (*n* = 5), group of MCAO 2 week (*n* = 5), and group of MCAO 2 month (*n* = 5). The sample size was reasonable based on the law of diminishing returns and the male rats were selected based on the published papers (Hao et al., 2018; Liu et al., 2019; Lin et al., 2020; Franke et al., 2021; Sun et al., 2022). Groups of MCAO 1 week, 2 week, and 2 month rats were subjected to ischemia stroke by the MCAO model as the previous studies (Hao et al., 2018; Lin et al., 2020; Sun et al., 2022). For details, they were anesthetized with 3 ml/kg of 10% chloral hydrate by intraperitoneal injection with heating and monitoring body temperature at 36 ± 0.5°C during the surgical procedures. Experimental rats were immobilized by a tooth holder and with binding of all limbs, followed by inserting poly- L-lysine coated nylon filament (2634A4, Cinontech Co., Ltd., Beijing, China) into the left middle cerebral artery through the left common carotid artery and internal carotid artery, to block blood flow to the left middle cerebral artery. Ninety minutes later, the filament was drawn out from the internal carotid artery to allow reperfusion. On the contrary, sham group rats with the sham operation were exposed to the internal carotid artery, external carotid artery, and common carotid artery without inserting filament. All rats successively underwent neurological examination and DCE-MRI, followed by removing the brain samples for immunofluorescence staining (Figure 1). All procedures performed in this study were approved by the Ethics Committee of Huashan Hospital, Fudan University, and performed by the National Institutes of Health Guide for the Care and Use of Laboratory Animal and reported by the ARRIVE guidelines (Animal Research: Reporting *in vivo* Experiments).

### Neurological examination

A neurological behavior scale of 0–20 scores was used to assess the sensorimotor function of the rats, including motility, gait disturbances, postural signs, lateral resistance, and limb placing (van der Zijden et al., 2008). Higher scores represent more neurological deficits.

### Magnetic resonance imaging and gadolinium-diethylenetriaminepentaacetic acid injection

The MRI measurements were performed in a 3.0-T horizontal magnet (Discovery MR750, GE Medical Systems, Milwaukee, WI, United States) with a 60-mm-diameter gradient coil (Magtron Inc., Jiangyin, China), as previous studies (Hao et al., 2018; Lin et al., 2020). T2-weighted MRI images were acquired by a fast spin-echo sequence with the following parameters: repetition time/echo time = 4,000/96 ms, field of view = 6 cm × 6 cm, matrix = 256 × 256, spacing = 0.2, slice thickness = 1.8 mm, spatial resolution = 0.24 mm × 0.24 mm × 1.8 mm, inter-slice distance = 2 mm, number of slices = 15, scan time = 3 min, echo train length = 15. High-resolution 3D T1-weighted MRI images were acquired by a gradient-recalled echo sequence with the following acquisition parameters: repetition time/echo time = 12/6 ms, field of view = 6 cm × 6 cm, matrix = 256 × 256, slice thickness = 1 mm, spatial resolution = 0.27 mm × 0.27 mm × 1 mm, slice thickness = 0.7 mm, flip angle = 15, scan time = 3.09 min.

The Gd-DTPA (Beilu^®^, China) injection was carried out as the previous study (Lin et al., 2020). Firstly, all rats were anesthesia with 10% chloral hydrate by intraperitoneal injection (3 ml/kg) and put in a stereotactic instrument. Next, the atlantooccipital membrane was exposed, followed by inserting a segment of polyethylene 10 tube and a 1 mm syringe needle into 2 mm deep in cisterna magna. Then, Gd-DTPA was injected at 2 ml/min to a total of 20 ml by a micro-syringe pump as a contrast agent. At the end of the injection, the needle remained in place for 5 additional minutes to prevent reflow. Finally, the polyethylene 10 tube and syringe needle were pulled out and the wound was glued with super glue. MRI images were acquired before Gd-DTPA injection (T2 and 3D T1) (baseline) and thereafter at 30 min, 1, 2, 3, 4, 5, and 6 h after Gd-DTPA injection (3D T1) (cisternography) (Lin et al., 2020). MRI anesthesia was also performed by intraperitoneal injection of chloral hydrate, and 0.2 ml chloral hydrate solution was injected every 1–1.5 h until MRI scanning was completed (baseline and cisternography). The T2 signal intensity was normalized by contralateral values as the ratio of T2SI (ipsilateral values/contralateral values) and was calculated three times to minimize measurement error. The T1 signal intensity of 6 h after Gd-DTPA injection was normalized by contralateral values as R1(6 h) [(ipsilateral T1 signal intensity of 6 h after Gd-DTPA injection-ipsilateral T1 signal intensity of baseline)/(contralateral T1 signal intensity of 6 h after Gd-DTPA injection- contralateral T1 signal intensity of baseline)] and was calculated three times to minimize measurement error.

The ischemic severity was checked by T2-weighted MRI 12–24 h after the operation. Thereafter, DCE-MRI with intrathecal Gd-DTPA administration was performed on the 1st day after the operation for the sham group, in the 1st week after the operation for the group of MCAO 1 week rats, in the 2nd week after the operation for the group of MCAO 2 week rats, as well as in the 2nd month after surgery for the group of MCAO 2 month rats (Figure 1).

### Immunofluorescence staining and analysis

Rats were perfused with phosphate buffer, followed by 4% paraformaldehyde, then brains were removed and post-fixed overnight at 4°C. After dehydration, wax leaching, embedding, and slicing, 10 serial coronary brain sections (thickness: 5 μm) of each rat were obtained at approximately −4.16 mm relative to the bregma, according to the Paxinos and Watson (2005). Well preserved sections were picked and immune-stained with anti-glial fibrillary acidic protein (anti-GFAP) (1:1,000, Abcam), anti-AQP4 (1:500, Abcam), anti-ionized calcium-binding adaptor molecule 1 (anti-Iba1) (1:200, Servicebio), anti-APP (1:1,000, Abcam) and 0.3% thioflavin S solution. Alexa Fluor 488- and CY3-conjugated antibodies (1:500, Servicebio) were used as secondary antibodies for immunofluorescence staining. Finally, sections were incubated with 4′,6-diamidino-2-phenylindole (1: 1,000, Sigma-Aldrich).

Immunofluorescence sections were scanned using a Vslide scanning microscope (Nikon, Chiyoda, Tokyo, Japan) with a ×20 primary objective. All images were acquired using constant scanning settings, and further semi-quantitatively analyzed to characterize the expression of those above immunofluorescence staining using Image J (National Institutes of Health, Bethesda, MD, USA).

Equivalent regions of interest were drawn on the ipsilateral (left) and contralateral (right) thalamus ventroposterior nucleus (VPN), and mean intensity and area% of all these markers of each region of interest were measured. Besides, AQP4 polarization was calculated as described in previous studies: the ratio of immunofluorescence intensity in high threshold and low threshold (Wang et al., 2012). An increased threshold was defined to exhibit AQP4 located in the whole brain parenchyma (which is named global AQP4), and a high threshold was defined to exhibit AQP4 only located in astrocytic end-feet (which is named perivascular AQP4). High polarization indicates higher perivascular AQP4 immunoreactivity relative to lower parenchymal AQP4 immunoreactivity, whereas reduced polarization indicates lower perivascular AQP4 immunoreactivity relative to higher parenchymal AQP4 immunoreactivity. All histological data were normalized by contralateral values as R2 [(ipsilateral values–contralateral values)/contralateral values] and were calculated twice to minimize measurement error.

### Statistical analysis

One-way analysis of variance (ANOVA) was performed for multiple group comparisons with *post-hoc* LSD tests performed for each of the two groups. Unpaired *t*-tests were performed for two groups comparison. The correlations between histological changes, MRI metrics, and functional scores were assessed using the Spearman correlation analysis. In statistical tests, differences were considered significant when *P* < 0.05. The statistical analysis was performed using Prism, version 8.3.0 (GraphPad Software Inc., La Jolla, CA, USA).

## Results

### The astrocyte activation in the ipsilateral thalamus was most obvious at the 2nd week after middle cerebral artery occlusion

**FIGURE 2:**
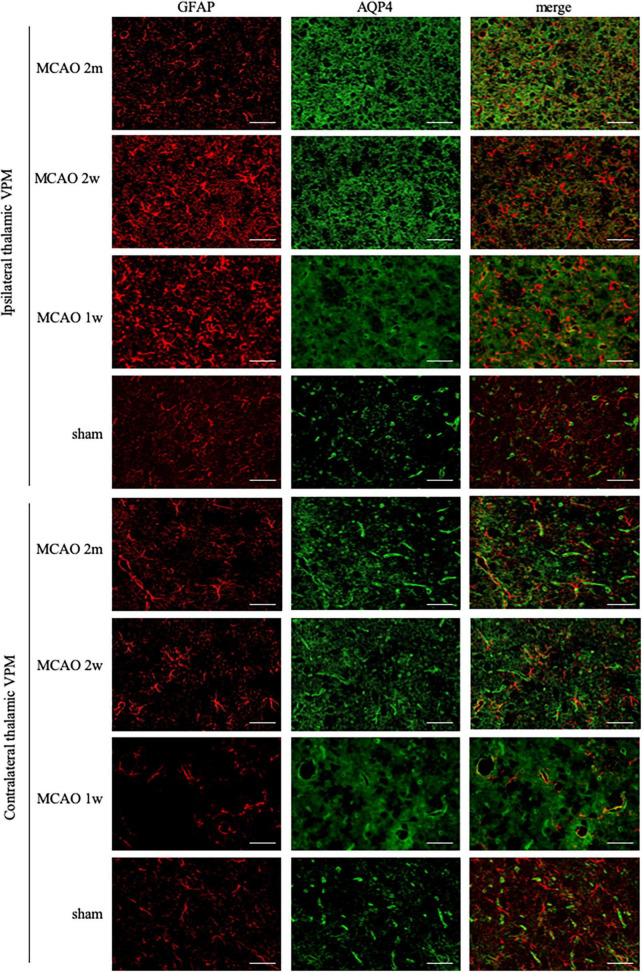
GFAP + AQP4 double-label staining in the thalamus VPN. Astrocyte activation (GFAP, red) was obvious in the ipsilateral thalamic VPN at the 1st week, the 2nd week, and the 2nd month after MCAO, compared with the contralateral thalamus VPN. At the 2nd week and the 2nd month after MCAO, most of the AQP4 (green) in the ipsilateral thalamus VPN had been dispersed in the brain parenchyma and the AQP4 polarization decreased. Bar = 50μm. GFAP, glial fibrillary acidic protein; AQP4, aquaporin 4; VPN, ventroposterior nucleus; MCAO, middle cerebral artery occlusion.

The GFAP levels between the ipsilateral and the contralateral thalamus VPN in MCAO 2 month group, MCAO 2 week group, and MCAO 1 week group had significant differences. Astrocyte was activated in the ipsilateral thalamic VPN at the 1st week, the 2nd week, and the 2nd month after MCAO (Figure 2).

**FIGURE 3:**
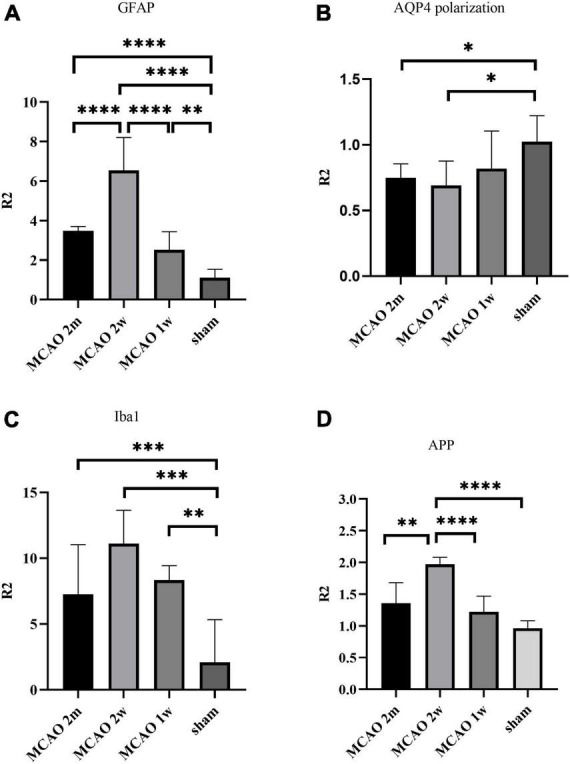
Quantitative analysis results of ratio (R2) in the thalamus VPN. The astrocyte and microglial activation **(A,C)** and the APP deposition **(D)** in the ipsilateral thalamus VPN were obvious, especially at the 2nd week after MCAO. The AQP4 polarization **(B)** in the ipsilateral thalamus VPN decreased, especially at the 2nd week and the 2nd month after MCAO. *P < 0.05; ^**^P < 0.01; ^***^P < 0.001; ^****^P < 0.0001. VPN, ventroposterior nucleus; APP, Beta-amyloid precursor protein; MCAO, middle cerebral artery occlusion; AQP4, aquaporin 4.

The R2_GFAP_ values of MCAO 2 month group, MCAO 2 week group and MCAO 1 week group were significantly higher than those of sham group (*P* < 0.0001, < 0.0001, = 0.0044). In addition, there were significant differences between MCAO 2 month group and MCAO 2 week group (*P* < 0.0001), MCAO 2 week group and MCAO 1 week group (*P* < 0.0001). At the 2nd week after MCAO, astrocyte activation in the ipsilateral thalamus VPN was most obvious, as shown in Figure 3A.

### The aquaporin-4 polarization rates in the ipsilateral thalamus decreased most at the 2nd week and the 2nd month after middle cerebral artery occlusion

The AQP4 staining in each group is shown in Figure 2. At the 2nd week and the 2nd month after MCAO, most of the AQP4 in the ipsilateral thalamus VPN had been dispersed in the brain parenchyma and the AQP4 polarization reduced significantly.

The R2_AQP4 polarization_ of the MCAO 2 week group and MCAO 2 month group was lower than that of the sham group (*P* = 0.0220, 0.0229). The R2_AQP4 polarization_ of the MCAO 1 week group was lower than that of the sham group, but it was not statistically significant. The AQP4 polarization rate in the ipsilateral thalamus VPN decreased, especially at the 2nd week and the 2nd month after MCAO, as shown in Figure 3B.

### The microglial activation in the ipsilateral thalamus was most obvious at the 2nd week after middle cerebral artery occlusion

**FIGURE 4:**
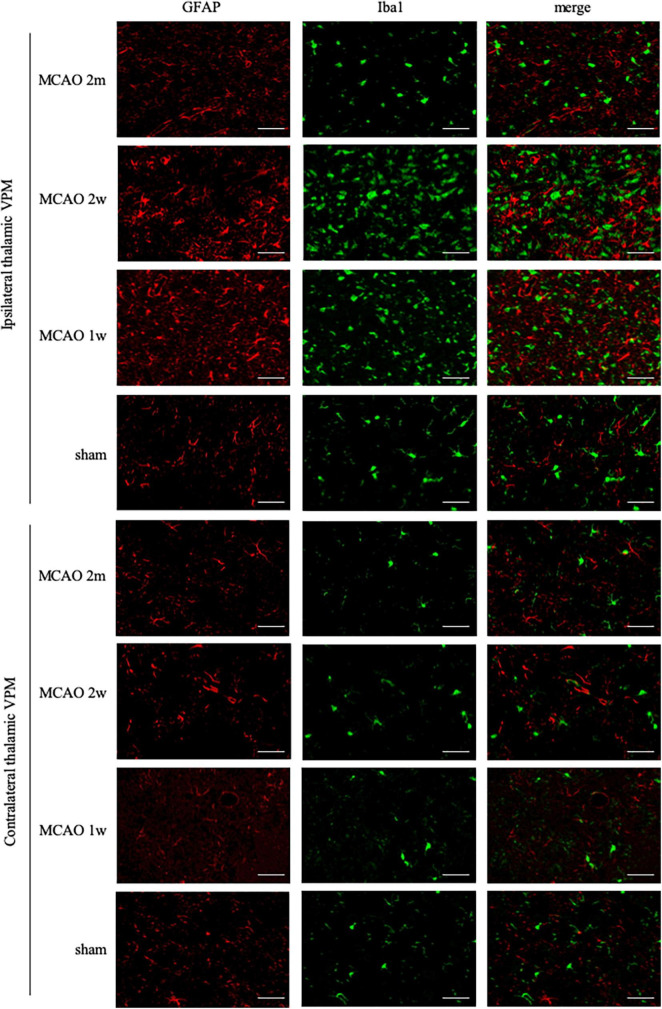
GFAP + Iba1 double-label staining in the thalamus VPN. Microglial activation (Iba1, green) was obvious in the ipsilateral thalamus VPN at the 1st week, the 2nd week, and the 2nd month after MCAO, compared with the contralateral thalamus VPN. Bar = 50 μm. GFAP, glial fibrillary acidic protein; Iba1, ionized calcium-binding adaptor molecule 1; VPN, ventroposterior nucleus; MCAO, middle cerebral artery occlusion.

Ionized calcium-binding adaptor molecule 1 is a specific staining marker of microglia. The expression of Iba1 levels between the ipsilateral and the contralateral thalamus VPN in the MCAO 2 month group, MCAO 2 week group, and MCAO 1 week group had significant differences. Microglial was activated in the ipsilateral thalamus VPN at the 1st week, the 2nd week, and the 2nd month after MCAO (Figure 4).

The R2_Iba1_ values of the MCAO 2 month group, MCAO 2 week group, and MCAO 1 week group were higher than the sham group (*P* = 0.0003, 0.0001, 0.0041). The microglial activation in the ipsilateral thalamus VPN was obvious, especially at the 2nd week after MCAO, as shown in Figure 3C.

### The beta-amyloid precursor protein deposition in the ipsilateral thalamus was most obvious at the 2nd week after middle cerebral artery occlusion

**FIGURE 5:**
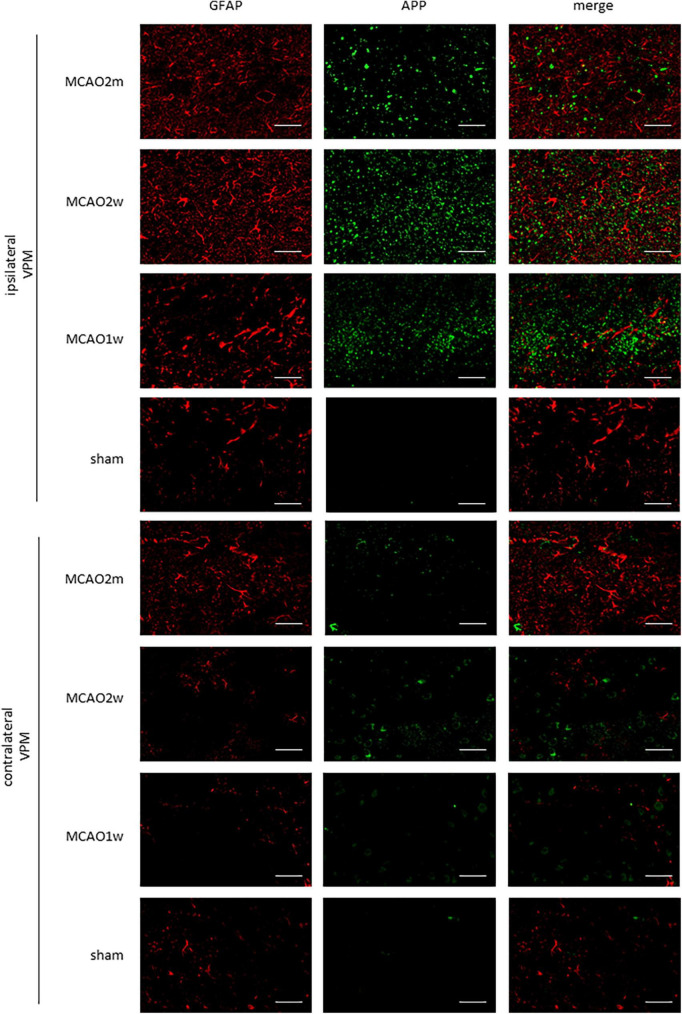
APP in the ipsilateral and contralateral thalamus VPN. There was little APP deposition in the ipsilateral thalamus of sham rats and the contralateral thalamus VPN of all rats. Bar = 50 μm. APP, beta-amyloid precursor protein; VPN, ventroposterior nucleus.

**FIGURE 6:**
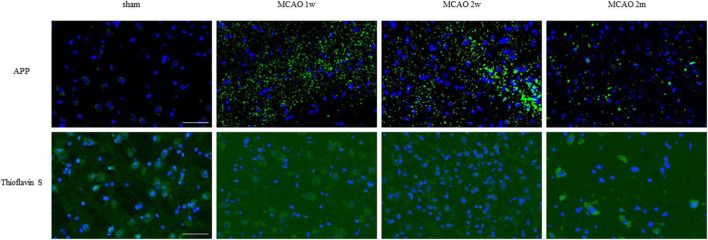
APP and mature amyloid plaque in the ipsilateral thalamus VPN. There was little APP deposition in the ipsilateral thalamus of sham rats. At the 1st week, the 2nd week, and the 2nd month after MCAO, APP deposition (green) showed a trend from less to more and gradually decreased in the ipsilateral thalamus VPN. Using Thioflavin S staining, no abnormal mature amyloid plaque was found in the ipsilateral thalamus VPN at each group after MCAO. Bar = 50 μm. APP, beta-amyloid precursor protein; VPN, ventroposterior nucleus; MCAO, middle cerebral artery occlusion.

The APP staining in the ipsilateral and contralateral thalamus VPN in each group is shown in Figure 5. There was little APP deposition in the thalamus of sham rats and the contralateral thalamus VPN of all rats. At the 1st week, the 2nd week, and the 2nd month after MCAO, APP deposition showed a trend from less to more, and then gradually decreased in the ipsilateral thalamus VPN (Figure 6).

The R2_APP_ value of the MCAO 1 week group was higher than the sham group, but the difference was not statistically significant. The R2_APP_ values of the MCAO 2 month and MCAO 2 week group were significantly higher than the sham group (*P* = 0.0063, < 0.0001), and the R2_APP_ value of the MCAO 2 week group was higher than the MCAO 1 week group. The APP deposition in the ipsilateral thalamus VPN was obvious, especially at the 2nd week after MCAO, as shown in Figure 3D.

Using Thioflavin S staining, no abnormal mature amyloid plaque was found in the thalamus VPN for each group after MCAO (Figure 6).

### The tendency of the beta-amyloid precursor protein deposition in the ipsilateral thalamus ventroposterior nucleus was consistent with the one of the contrast medium retention in dynamic contrast-enhanced MRI

**FIGURE 7:**
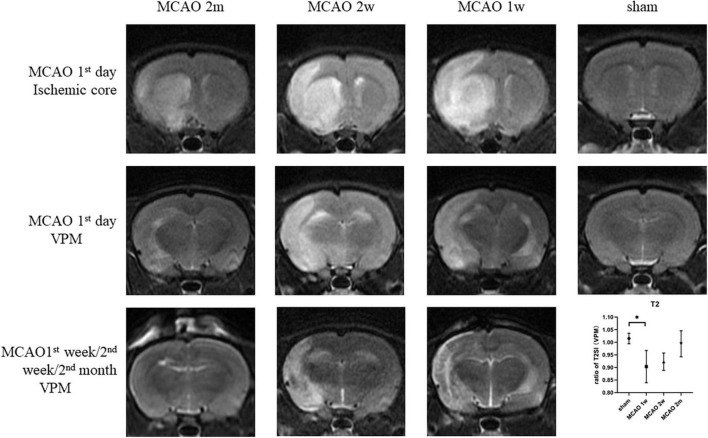
The T2SI of the ischemic cores and the ipsilateral thalamus VPN. On the 1st day after MCAO, the ischemic lesions were high signal at T2-weighted MRI for MCAO 2 month group, MCAO 2 week group, and MCAO 1 week group. The ratio of T2SI of the ipsilateral thalamus VPN touched bottom at the 1st week after MCAO and gradually recovered at the 2nd week and the 2nd month. T2SI, T2 signal intensity; VPN, ventroposterior nucleus; MCAO, middle cerebral artery occlusion.

On the 1st day after MCAO, the ischemic cores were high signal at T2-weighted MRI for MCAO 2 month group, MCAO 2 week group, and MCAO 1 week group. The ratio of T2SI of the ipsilateral thalamus VPN touched its bottom at the 1st week after MCAO and gradually recovered at the 2nd week and the 2nd month (Figure 7).

**FIGURE 8:**
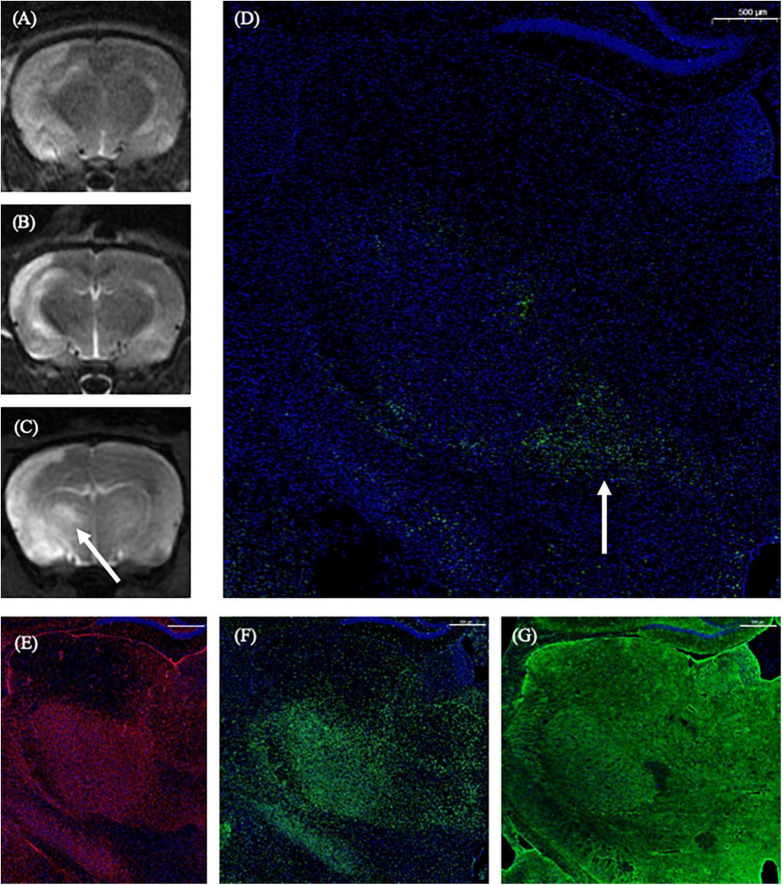
The correspondence between DCE-MRI and immunofluorescence staining results in the thalamus VPN. On the 1st day and the 2nd week after MCAO, the ipsilateral thalamus VPN was almost normal at T2-weighted MRI **(A,B)**. At the 2nd week after MCAO, the abnormal aggregation area of contrast medium at 3D T1-weighted MRI **(C)** included almost all the ipsilateral thalamus VPN, which was roughly consistent with the astrocytes and microglia activation **(E,F)**, and the AQP4 depolarization **(G)**. The tendency of the APP deposition **(D, arrow)** was consistent with the one of the contrast medium retention in DCE-MRI **(C, arrow)** in the ipsilateral thalamus VPN. Bar = 500 μm. DCE-MRI, dynamic contrast-enhanced MRI; MCAO, middle cerebral artery occlusion; VPN, ventroposterior nucleus; AQP4, aquaporin 4; APP, Beta-amyloid precursor protein.

On the 1st day and the 2nd week after MCAO, the ipsilateral thalamus VPN was almost normal at T2-weighted MRI. At the 2nd week after MCAO, the abnormal aggregation area of the contrast medium included almost all the ipsilateral thalamic VPN, which was roughly consistent with the area of astrocytes and microglia proliferation and the AQP4 depolarization. The lateral part of the ipsilateral thalamus VPN was more obvious than the central small cell part. However, for the APP deposition, small cells in the central part of the ipsilateral thalamus VPN were more obvious than in the lateral part. The tendency of the APP deposition in the ipsilateral thalamus VPN was consistent with the one of the contrast medium retention in DCE-MRI, but the location was not the same (Figure 8).

There was no correlation between histological changes (R2 of GFAP, AQP4 polarization, Iba1, and APP) and MRI metrics [the ratio of T2SI and R1(6 h)] in all four groups (*P* > 0.05 for all).

### The neurological function deteriorated most at the 1st week and the 2nd week after middle cerebral artery occlusion

**FIGURE 9:**
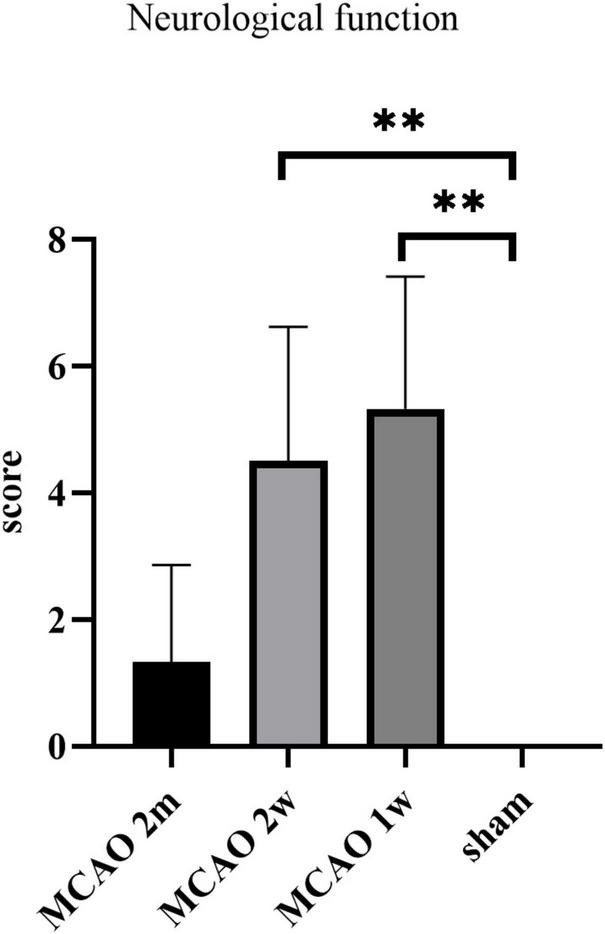
The neurological function scores. The neurological function deteriorated at the 1st week and the 2nd week after MCAO, and gradually recovered at the 2nd month. ^**^P < 0.01. MCAO, middle cerebral artery occlusion.

The neurological function score was the highest in MCAO 1 week group, which was significantly higher than the sham group (*P* = 0.0037). The score of the MCAO 2 week group was significantly higher than the sham group (*P* = 0.0013), but lower than MCAO 1 week group. The score of the MCAO 2 month group was lower than MCAO 2 week group and higher than the sham group. In other words, the neurological function deteriorated most at the 1st week and the 2nd week after MCAO, and gradually recovered at the 2nd month, but it was still lower than the healthy level (Figure 9).

There was no correlation between neurological function score and histological changes (R2 of GFAP, AQP4 polarization, Iba1, and APP), as well as MRI metrics [the ratio of T2SI and R1(6 h)] in the MCAO 2 month group, MCAO 2 week group, and MCAO 1 week group (*P* > 0.05 for all).

## Discussion

This study investigated the structural and functional dynamic changes of the impaired glymphatic system in the thalamus, secondary degeneration area after ischemic stroke in rats by pathology and MRI. Our results indicated that the astrocyte and microglial activation, the AQP4 depolarization, and the APP deposition in the thalamus were obvious, especially at the 2nd week after MCAO. Although there was no correlation between histological changes and MRI metrics statistically, the tendency of the APP deposition was in line with the one of the contrast medium retention in DCE-MRI. In general, this study highlighted the glymphatic system in the thalamus was severely impaired at the 2nd week after MCAO, and may be revealed by DCE-MRI.

As we all know, the brain has an extremely high density of cells, with a high metabolic activity that is disproportionate to its size, and which produces a large number of metabolites. The brain may have a different metabolic-clearing pathway than other tissues. The discovery of the perivascular space and the glymphatic system provides a solution for this contradiction. AQP4 is an important bridge in the glymphatic system (Rasmussen et al., 2018). CSF and ISF are mainly exchanged through AQP4, but the specific exchange mechanism between the two fluids still requires further study. In the physiological condition, the glymphatic system facilitates the clearance of metabolic wastes from the brain and helps maintain homeostasis of the central nervous system (Rasmussen et al., 2018; Nedergaard and Goldman, 2020; Zhou et al., 2021).

Interestingly, when damage to the central nervous system occurs, such as ischemic stroke, the flow in the glymphatic system is often significantly decreased for some time, and the degree and speed of recovery of the glymphatic system correlate with the prognosis of stroke (Rasmussen et al., 2018; Feng et al., 2020; Zhou et al., 2021). As an important part of the glymphatic system, the AQP4 has been shown to change the location from the end feet of perivascular astrocytes to other places after stroke. This was AQP4 depolarization (Troili et al., 2020). Astrocytes are also important components of the glymphatic system. This suggests that astrocytic hyperplasia, accompanied by the loss of AQP4 polarization, may impair the glymphatic system. Our data are in agreement with these findings. The glymphatic pathway has emerged as a key mechanism of clearance of APP. Since a remarkable amount of APP is eliminated through the glymphatic pathway, impaired glymphatic clearance could be implicated in the development and progression of stroke. Interestingly, APP has a detrimental effect on the glymphatic clearance, thus potentially leading to a deleterious feedback loop toward further impaired drainage and central nervous system injury (Troili et al., 2020).

Gaberel et al. (2014) used DCE-MRI to investigate the impact of different stroke subtypes on the glymphatic system and found that glymphatic perfusion remained impaired at 3 h poststroke and appeared normal at 24 h after stroke onset. Our result is inconsistent with it. There may be some reasons. Firstly, embolic ischemic stroke was induced in mice by mechanically promoting embolization of Ferric chloride (FeCl3)-triggered thrombi (30% m/v, 1 min application) to the middle cerebral artery by Gaberel et al. (2014) while poly- L-lysine coated nylon filament (2634A4, Cinontech Co., Ltd., Beijing, China) inserted into the middle cerebral artery in our study. The animal model in our study is more conventional. Secondly, in the Gaberel’s study, all the performed mice had two angiographies which revealed that the middle cerebral artery was repermeabilized in all mice 24 h after stroke onset, while we did not perform angiography and cannot confirm whether the middle cerebral artery was repermeabilized in all rats. It is one of the areas for improvement in our future study. Thirdly, immunofluorescence staining and analysis, including astrocyte activation, AQP4 polarization, microglial activation, and APP deposition were performed in our study, and all these histological changes were abnormal until 2 months after MCAO, so we inferred that the glymphatic system remain impaired in structure until 2 months poststroke. While only DCE-MRI was used in the Gaberel’s study and the result of “glymphatic perfusion appeared normal at 24 h after stroke onset” focused on function.

Microglia in the activated state engulfs abnormal substances in the brain parenchyma. Our finding agrees with these reporters that harmful substances such as APP accumulate in the thalamus VPN, which advances the microglia activation (Feng et al., 2020). Microglial activation accelerates the elimination of harmful substances in the brain parenchyma, therefore reducing the deposition of APP to a certain extent and playing a neuroprotective role. Our results suggest that microglia may play a role in promoting the recovery of secondary degeneration after ischemic stroke.

The impaired glymphatic system decreases the clearance efficiency of soluble proteins in the brain parenchyma, and the persistence of glymphatic system dysfunction eventually leads to the deposition of harmful proteins in the brain parenchyma. By comprehensive comparison with the results of DCE-MRI and immunofluorescence staining at 2nd week after MCAO, we found that the abnormal aggregation of contrast medium occurred in the whole ipsilateral thalamus VPN region, and the astrocytes and microglia proliferation and the AQP4 depolarization were also in the whole ipsilateral thalamus VPN region. However, the APP deposition was more obvious in the central part of the thalamus VPN, that was, the small cell part. We speculate that the thalamus VPN small cell part is the initial concentration part of the thalamus striatum pathway fiber bundle, which is the more observable site of retrograde secondary degeneration along the axon (Iwai et al., 2015). The APP deposition is more noticeable in the thalamus VPN small cell part, which may be related to the pathological mechanism of retrograde degeneration. While our results also displayed that there was no correlation between histological changes and MRI metrics in all four groups statistically. It may be due to some reasons such as the complexity of MRI signal formation and limitations of MRI signal intensity evaluation. The application of radiomics may get positive results in future study.

We found that the neurological function deteriorated most at the 1st week and the 2nd week after MCAO, and gradually recovered at the 2nd month. This performance was consistent with the tendency of the APP deposition in the thalamus. Our finding raises the possibility that the harmful substances deposition in the thalamus after ischemic stroke may have a certain effect on the neurological function. However, there was no correlation between neurological function scores and histological changes in MCAO 2 month group, MCAO 2 week group, and MCAO 1 week group statistically. Expanding the sample size may lead to positive results in future study.

As for the failure of Thioflavin S staining to show mature amyloid plaques in the thalamus after MCAO in this study, it is inconsistent with the earlier studies (Bussière et al., 2004; Shin et al., 2021). There may be two reasons. Firstly, transgenic mice-heterozygous transgenic mice expressing mutated hAPP (PDAP mice) and 5 × FAD mice expressing β-sheet-rich proteins other than Aβ were used in the earlier studies, while Sprague Dawley rates were used in our experiment. Secondly, it also may be due to other reasons such as plaque formation time and plaque subtype (type 1, type 2a, type 2b, and type 2c). This needs further study.

The present study has several limitations. Firstly, we did not study the functional and pathophysiological changes of the glymphatic system in the thalamus in the hyperacute stage, in the acute stage or chronic stage after MCAO, and also in the peripheral infarct. Secondly, R2 was not a fair metric to longitudinally assess the AQP4 change since it was calculated by (ipsilateral values − contralateral values)/contralateral values and both sides changed over time. One way to confirm this assessment is to compare the change in the thalamus to other brain regions, such as the cortex. But both our previous experiments and this experiment showed that more than 2/3 of the ipsilateral cortex was also affected by ischemia. This calculation method will be improved in future experiments. Thirdly, it was reported that significant secondary neuronal loss and reactive gliosis occur in the thalamus and hippocampus after MCAO in non-human primates (Ouyang et al., 2020). Maybe a non-human primate model is needed in the future to improve the clinical transformation of experimental results. Fourthly, we did not try to intervene in the key proteins or molecules of the glymphatic system to explore the changes after the intervention and whether the intervention contributes to the recovery of neurological function after ischemic stroke. Fifthly, recently MRI-visible enlarged perivascular space has been proposed as an MRI biomarker of the glymphatic pathway (Hilal et al., 2018; Liu et al., 2018; Jochems et al., 2020; Penton et al., 2020; Troili et al., 2020). However, it remains unclear whether enlarged perivascular space reflects glymphatic stasis or rather increased capacity of the perivascular space to drain potentially neurotoxic metabolites. Additional studies are necessary to confirm the clinical significance of enlarged perivascular space. Sixthly, the images under study, as known, could be affected by uncertainties and/or imprecision. In these cases, fuzzy image preprocessor, as scientific literature suggests, has to be exploited (Versaci et al., 2015). Such a tool may be used in our future studies. Seventhly, there was no condition to imply gas anesthesia, *in vivo* two-photon imaging, angiography, and Ki-67 staining in this study. All of these are the areas for improvement in our future study.

All in all, this study investigated the structural and functional dynamic changes of the impaired glymphatic system in the thalamus, secondary degeneration area after ischemic stroke in rats by pathology and MRI. The major strength of this study was finding the glymphatic system was severely impaired at the 2nd week after MCAO. Further studies are warranted to investigate the indirect mechanism and in-depth direct mechanism of the impaired glymphatic system at the molecular level, and at the same time, to explore using the internal features of multi-modal images to predict neurological scores at the radiomics level. Our study may provide the theoretical basis for making a thorough inquiry of the mechanism of brain injury after stroke and clinical treatment, such as broadening the treatment time window to the 2nd week, exploring new targets for the treatment of secondary degenerative areas, then eventually help to improve the prognosis of ischemic stroke. Our study also helps readers appreciate the importance of DCE-MRI in animal research and prompts that DCE-MRI is a potential clinically acceptable approach that can be used to visualize and evaluate the glymphatic system.

## Data availability statement

The raw data supporting the conclusions of this article will be made available by the authors, without undue reservation.

## Ethics statement

This animal study was reviewed and approved by the Ethics Committee of Huashan Hospital, Fudan University.

## Author contributions

CL, LL, ZY, XF, and YY were responsible for the study concept and the design. CL, LL, CS, and XH collected the data. CL, LL, CS, XH, LY, XZ, and JT analyzed the data and conducted the statistical analysis. CL and LL wrote the manuscript. ZY, XF, and YY supervised the manuscript and provided technical or information support. All authors approved the final version of the manuscript.
